# 1-(3-*tert*-Butyl-4-hy­droxy­phen­yl)ethanone

**DOI:** 10.1107/S1600536810027339

**Published:** 2010-07-17

**Authors:** Hua-Ming Miao, Gui-Long Zhao, Hua Shao, Jian-Wu Wang

**Affiliations:** a School of Chemistry and Chemical Engineering, Shandong University, Jinan 250100, People’s Republic of China; b Tianjin Key Laboratory of Molecular Design and Drug Discovery, Tianjin Institute of Pharmaceutical Research, Tianjin 300193, People’s Republic of China

## Abstract

The title compound, C_12_H_16_O_2_, is approximately planar (r.m.s. deviation = 0.030 Å), apart from two methyl groups of the *tert*-butyl unit [deviations of the C atoms = 1.140 (2) and −1.367 (1) Å]. In the crystal, inter­molecular O—H⋯O hydrogen bonds link the mol­ecules into hexa­meric rings with *R*_6_^6^(48) graph-set motifs.

## Related literature

For details of the biological activity of the PAR-1 antagonist, see: Chackalamannil (2006 ▶); Shimomura *et al.* (2006 ▶). For bond-length data, see: Allen *et al.* (1987 ▶).
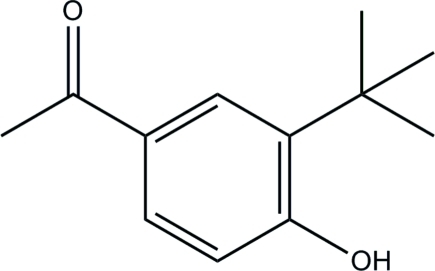


## Experimental

### 

#### Crystal data

C_12_H_16_O_2_
*M* *_r_* = 192.25
Trigonal, 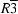

*a* = 24.019 (3) Å
*c* = 9.999 (2) Å
*V* = 4995.8 (14) Å^3^
*Z* = 18
Mo *K*α radiation
μ = 0.08 mm^−1^
*T* = 113 K
0.20 × 0.18 × 0.14 mm

#### Data collection

Rigaku Saturn CCD diffractometer
Absorption correction: multi-scan (*CrystalClear*; Rigaku, 2005 ▶) *T*_min_ = 0.985, *T*_max_ = 0.989
12180 measured reflections
1950 independent reflections
1733 reflections with *I* > 2σ(*I*)
*R*_int_ = 0.034

#### Refinement

*R*[*F* ^2^ > 2σ(*F* ^2^)] = 0.039
*wR*(*F* ^2^) = 0.110
*S* = 1.03
1950 reflections
133 parameters
H-atom parameters constrained
Δρ_max_ = 0.24 e Å^−3^
Δρ_min_ = −0.18 e Å^−3^

### 

Data collection: *CrystalClear* (Rigaku, 2005 ▶); cell refinement: *CrystalClear*; data reduction: *CrystalClear*; program(s) used to solve structure: *SHELXTL* (Sheldrick, 2008 ▶); program(s) used to refine structure: *SHELXTL*; molecular graphics: *SHELXTL*; software used to prepare material for publication: *SHELXTL*.

## Supplementary Material

Crystal structure: contains datablocks I, global. DOI: 10.1107/S1600536810027339/hb5546sup1.cif

Structure factors: contains datablocks I. DOI: 10.1107/S1600536810027339/hb5546Isup2.hkl

Additional supplementary materials:  crystallographic information; 3D view; checkCIF report

## Figures and Tables

**Table 1:** Hydrogen-bond geometry (Å, °)

*D*—H⋯*A*	*D*—H	H⋯*A*	*D*⋯*A*	*D*—H⋯*A*
O1—H1⋯O2^i^	0.84	1.83	2.6624 (12)	171

Symmetry code: (i) 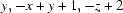.

**Fig. 1.**
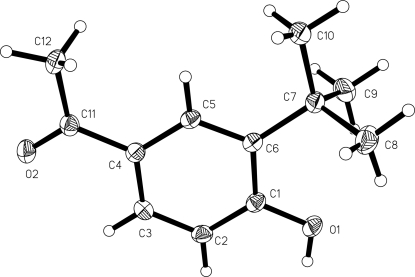
View of (I), with displacement ellipsoids drawn at the 40% probability level.

**Fig. 2.**
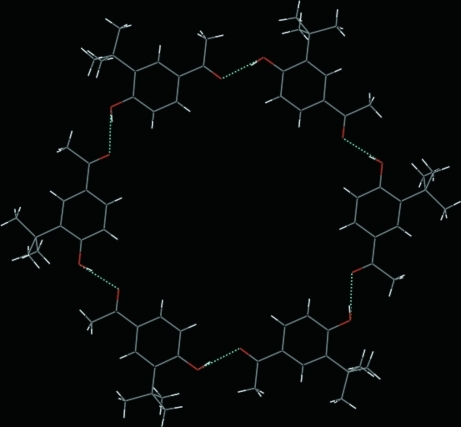
View of the hexameric ring in the crystal of (I).

## supplementary crystallographic information

### Comment

PAR-1 antagonist is a kind of new anti-platelet agents in the antithrombotic area for the treat of artery coronary syndrome (Chackalamannil, 2006). The title compound is prepared when the well established PAR-1 antagonist E-5555 was synthesized as positive control during the development of our own PAR-1 antagonists (Shimomura *et al.*, 2006).

In title compound, C_12_H_16_O_2_, bond lengths are normal ((Allen *et al.*, 1987)). Intermolecular interactions O—H···O hydrogen bonds link the moleculars into hexamer.

### Experimental

A dried 500-ml round-bottomed flask was charged with 21.33 g (0.160 mol, 1.2 eq) of anhydrous aluminium chloride and 400 ml of dried toluene, and the resulting yellow slurry was stirred and cooled to -35°C followed by dropwise addition of 20.0 g (0.133 mol, 1.0 eq) of 2-(*tert*-butyl)phenol dissolved in 20 ml of dried toluene. To the yellow clear solution obtained above was added dropwise 12.56 g (0.160 mol, 1.2 eq) of acetyl chloride dissolved in 20 ml of dried toluene, and after addition the resulting mixture (a yellow clear solution) was stirred at this temperature until all the starting material was consumed almost completely as indicated by TLC analysis (typical 2–3 h). The reaction mixture was slowly poured into 500 ml of stirred ice-water with great care, and the resulting mixture was stirred. The organic phase was separated and the aqueous phase was exacted with three 100-ml portions of ethyl acetate. The combined exacts were washed with brine to pH = 7, dried over sodium sulfate and evaporated on a rotary evaporator to afford the crude product as colorless crystals, which was triturated with ethyl acetate/petroleum ether (1/30) to afford the pure product as colorless crystals. Colourless blocks of (I) were obtained *via* slow evaporation at room temperature of a solution of the pure title compound in ethyl acetate/petroleum ether (1/30).

### Refinement

All H atoms were found on difference maps, with C—H = 0.95 or 0.98 and O—H = 0.84 Å and included in the final cycles of refinement using a riding model, with *U*_iso_(H) = 1.2*U*_eq_(C) for aryl H atoms and 1.5*U*_eq_(C, O) for the methyl and hydroxy H atoms.

### Crystal data

**Table:**

C_12_H_16_O_2_	*D*_x_ = 1.150 Mg m^−^^3^
*M**_r_* = 192.25	Mo *K*α radiation, λ = 0.71073 Å
Trigonal, *R*3	Cell parameters from 5019 reflections
Hall symbol: -R 3	θ = 2.3–27.9°
*a* = 24.019 (3) Å	µ = 0.08 mm^−^^1^
*c* = 9.999 (2) Å	*T* = 113 K
*V* = 4995.8 (14) Å^3^	Block, colorless
*Z* = 18	0.20 × 0.18 × 0.14 mm
*F*(000) = 1872	

### Data collection

**Table:**

Rigaku Saturn CCD diffractometer	1950 independent reflections
Radiation source: rotating anode	1733 reflections with *I* > 2σ(*I*)
confocal	*R*_int_ = 0.034
Detector resolution: 7.31 pixels mm^-1^	θ_max_ = 25.0°, θ_min_ = 1.7°
ω and φ scans	*h* = −21→28
Absorption correction: multi-scan (*CrystalClear*; Rigaku, 2005)	*k* = −28→28
*T*_min_ = 0.985, *T*_max_ = 0.989	*l* = −11→11
12180 measured reflections	

### Refinement

**Table:**

Refinement on *F*^2^	Secondary atom site location: difference Fourier map
Least-squares matrix: full	Hydrogen site location: inferred from neighbouring sites
*R*[*F*^2^ > 2σ(*F*^2^)] = 0.039	H-atom parameters constrained
*wR*(*F*^2^) = 0.110	*w* = 1/[σ^2^(*F*_o_^2^) + (0.0723*P*)^2^ + 1.6812*P*] where *P* = (*F*_o_^2^ + 2*F*_c_^2^)/3
*S* = 1.03	(Δ/σ)_max_ = 0.002
1950 reflections	Δρ_max_ = 0.24 e Å^−^^3^
133 parameters	Δρ_min_ = −0.18 e Å^−^^3^
0 restraints	Extinction correction: *SHELXTL* (Sheldrick, 2008), Fc^*^=kFc[1+0.001xFc^2^λ^3^/sin(2θ)]^-1/4^
Primary atom site location: structure-invariant direct methods	Extinction coefficient: 0.0080 (7)

### Special details

**Table:**

Geometry. All e.s.d.'s (except the e.s.d. in the dihedral angle between two l.s. planes) are estimated using the full covariance matrix. The cell e.s.d.'s are taken into account individually in the estimation of e.s.d.'s in distances, angles and torsion angles; correlations between e.s.d.'s in cell parameters are only used when they are defined by crystal symmetry. An approximate (isotropic) treatment of cell e.s.d.'s is used for estimating e.s.d.'s involving l.s. planes.
Refinement. Refinement of *F*^2^ against ALL reflections. The weighted *R*-factor *wR* and goodness of fit *S* are based on *F*^2^, conventional *R*-factors *R* are based on *F*, with *F* set to zero for negative *F*^2^. The threshold expression of *F*^2^ > σ(*F*^2^) is used only for calculating *R*-factors(gt) *etc*. and is not relevant to the choice of reflections for refinement. *R*-factors based on *F*^2^ are statistically about twice as large as those based on *F*, and *R*- factors based on ALL data will be even larger.

### Fractional atomic coordinates and isotropic or equivalent isotropic displacement parameters (Å^2^)

**Table:**

	*x*	*y*	*z*	*U*_iso_*/*U*_eq_	
O1	0.57494 (4)	0.80946 (4)	0.95585 (9)	0.0304 (3)	
H1	0.6049	0.8470	0.9716	0.046*	
O2	0.72860 (4)	0.66048 (4)	0.99295 (9)	0.0296 (3)	
C1	0.59747 (6)	0.76811 (5)	0.96507 (12)	0.0230 (3)	
C2	0.66314 (6)	0.79128 (5)	0.98477 (12)	0.0248 (3)	
H2	0.6918	0.8362	0.9924	0.030*	
C3	0.68665 (6)	0.74960 (6)	0.99315 (11)	0.0241 (3)	
H3	0.7315	0.7658	1.0036	0.029*	
C4	0.64445 (5)	0.68348 (5)	0.98621 (11)	0.0215 (3)	
C5	0.57896 (5)	0.66128 (5)	0.96739 (11)	0.0214 (3)	
H5	0.5504	0.6162	0.9639	0.026*	
C6	0.55354 (5)	0.70158 (5)	0.95352 (11)	0.0213 (3)	
C7	0.48199 (6)	0.67589 (6)	0.92360 (13)	0.0272 (3)	
C8	0.45154 (6)	0.69730 (7)	1.03216 (15)	0.0386 (4)	
H8A	0.4588	0.6840	1.1201	0.058*	
H8B	0.4052	0.6773	1.0159	0.058*	
H8C	0.4711	0.7442	1.0299	0.058*	
C9	0.47547 (7)	0.70091 (7)	0.78650 (14)	0.0395 (4)	
H9A	0.4980	0.7480	0.7879	0.059*	
H9B	0.4299	0.6843	0.7666	0.059*	
H9C	0.4943	0.6864	0.7175	0.059*	
C10	0.44439 (6)	0.60217 (6)	0.91906 (15)	0.0373 (4)	
H10A	0.4622	0.5871	0.8489	0.056*	
H10B	0.3991	0.5872	0.8997	0.056*	
H10C	0.4479	0.5851	1.0057	0.056*	
C11	0.67018 (6)	0.63918 (6)	0.99456 (11)	0.0236 (3)	
C12	0.62460 (6)	0.56802 (6)	1.00527 (13)	0.0297 (3)	
H12A	0.6491	0.5459	1.0191	0.045*	
H12B	0.5995	0.5524	0.9227	0.045*	
H12C	0.5955	0.5593	1.0811	0.045*	

### Atomic displacement parameters (Å^2^)

**Table:**

	*U*^11^	*U*^22^	*U*^33^	*U*^12^	*U*^13^	*U*^23^
O1	0.0294 (5)	0.0171 (4)	0.0472 (6)	0.0134 (4)	−0.0021 (4)	0.0000 (4)
O2	0.0260 (5)	0.0321 (5)	0.0356 (5)	0.0182 (4)	−0.0016 (4)	0.0001 (4)
C1	0.0287 (7)	0.0206 (6)	0.0224 (6)	0.0144 (5)	0.0003 (5)	0.0014 (5)
C2	0.0263 (6)	0.0177 (6)	0.0270 (6)	0.0083 (5)	−0.0023 (5)	−0.0010 (5)
C3	0.0223 (6)	0.0261 (6)	0.0230 (6)	0.0113 (5)	−0.0026 (5)	−0.0003 (5)
C4	0.0251 (6)	0.0224 (6)	0.0186 (6)	0.0131 (5)	−0.0005 (4)	0.0003 (4)
C5	0.0249 (6)	0.0184 (6)	0.0210 (6)	0.0109 (5)	0.0003 (5)	0.0004 (4)
C6	0.0240 (6)	0.0200 (6)	0.0204 (6)	0.0115 (5)	0.0008 (5)	0.0002 (4)
C7	0.0230 (6)	0.0218 (6)	0.0385 (7)	0.0126 (5)	−0.0013 (5)	−0.0007 (5)
C8	0.0294 (7)	0.0303 (7)	0.0577 (9)	0.0163 (6)	0.0115 (6)	0.0026 (6)
C9	0.0362 (8)	0.0395 (8)	0.0461 (8)	0.0215 (7)	−0.0152 (6)	−0.0038 (6)
C10	0.0220 (7)	0.0252 (7)	0.0626 (9)	0.0102 (6)	−0.0031 (6)	−0.0050 (6)
C11	0.0271 (7)	0.0288 (7)	0.0188 (6)	0.0169 (5)	−0.0015 (5)	−0.0003 (5)
C12	0.0310 (7)	0.0257 (7)	0.0385 (7)	0.0188 (6)	−0.0017 (5)	0.0013 (5)

### Geometric parameters (Å, °)

**Table:**

O1—C1	1.3512 (14)	C7—C10	1.5343 (17)
O1—H1	0.8400	C7—C9	1.5359 (19)
O2—C11	1.2299 (14)	C8—H8A	0.9800
C1—C2	1.3995 (17)	C8—H8B	0.9800
C1—C6	1.4121 (16)	C8—H8C	0.9800
C2—C3	1.3761 (17)	C9—H9A	0.9800
C2—H2	0.9500	C9—H9B	0.9800
C3—C4	1.3944 (17)	C9—H9C	0.9800
C3—H3	0.9500	C10—H10A	0.9800
C4—C5	1.3983 (16)	C10—H10B	0.9800
C4—C11	1.4762 (16)	C10—H10C	0.9800
C5—C6	1.3857 (16)	C11—C12	1.5034 (17)
C5—H5	0.9500	C12—H12A	0.9800
C6—C7	1.5372 (16)	C12—H12B	0.9800
C7—C8	1.5342 (18)	C12—H12C	0.9800
			
C1—O1—H1	109.5	C7—C8—H8B	109.5
O1—C1—C2	120.22 (10)	H8A—C8—H8B	109.5
O1—C1—C6	118.61 (10)	C7—C8—H8C	109.5
C2—C1—C6	121.16 (10)	H8A—C8—H8C	109.5
C3—C2—C1	120.68 (10)	H8B—C8—H8C	109.5
C3—C2—H2	119.7	C7—C9—H9A	109.5
C1—C2—H2	119.7	C7—C9—H9B	109.5
C2—C3—C4	119.76 (10)	H9A—C9—H9B	109.5
C2—C3—H3	120.1	C7—C9—H9C	109.5
C4—C3—H3	120.1	H9A—C9—H9C	109.5
C3—C4—C5	118.66 (10)	H9B—C9—H9C	109.5
C3—C4—C11	119.35 (10)	C7—C10—H10A	109.5
C5—C4—C11	121.96 (10)	C7—C10—H10B	109.5
C6—C5—C4	123.49 (10)	H10A—C10—H10B	109.5
C6—C5—H5	118.3	C7—C10—H10C	109.5
C4—C5—H5	118.3	H10A—C10—H10C	109.5
C5—C6—C1	116.18 (10)	H10B—C10—H10C	109.5
C5—C6—C7	122.26 (10)	O2—C11—C4	120.07 (11)
C1—C6—C7	121.54 (10)	O2—C11—C12	120.31 (10)
C8—C7—C10	107.67 (10)	C4—C11—C12	119.62 (10)
C8—C7—C9	109.96 (11)	C11—C12—H12A	109.5
C10—C7—C9	107.98 (11)	C11—C12—H12B	109.5
C8—C7—C6	110.64 (10)	H12A—C12—H12B	109.5
C10—C7—C6	111.33 (10)	C11—C12—H12C	109.5
C9—C7—C6	109.21 (10)	H12A—C12—H12C	109.5
C7—C8—H8A	109.5	H12B—C12—H12C	109.5
			
O1—C1—C2—C3	−179.44 (11)	C2—C1—C6—C7	−176.33 (11)
C6—C1—C2—C3	0.13 (18)	C5—C6—C7—C8	122.54 (12)
C1—C2—C3—C4	−2.04 (18)	C1—C6—C7—C8	−59.10 (15)
C2—C3—C4—C5	1.59 (17)	C5—C6—C7—C10	2.85 (16)
C2—C3—C4—C11	179.67 (10)	C1—C6—C7—C10	−178.79 (11)
C3—C4—C5—C6	0.82 (18)	C5—C6—C7—C9	−116.30 (12)
C11—C4—C5—C6	−177.21 (10)	C1—C6—C7—C9	62.07 (14)
C4—C5—C6—C1	−2.62 (17)	C3—C4—C11—O2	−7.86 (17)
C4—C5—C6—C7	175.82 (11)	C5—C4—C11—O2	170.15 (11)
O1—C1—C6—C5	−178.30 (10)	C3—C4—C11—C12	172.03 (10)
C2—C1—C6—C5	2.13 (17)	C5—C4—C11—C12	−9.95 (17)
O1—C1—C6—C7	3.25 (17)		

### Hydrogen-bond geometry (Å, °)

**Table:**

*D*—H···*A*	*D*—H	H···*A*	*D*···*A*	*D*—H···*A*
O1—H1···O2^i^	0.84	1.83	2.6624 (12)	171

Symmetry codes: (i) *y*, −*x*+*y*+1, −*z*+2.
